# Multi-omics integration reveals organ-specific biosynthesis of flavonoids and terpenoids in the medicinal plant *Bidens alba*

**DOI:** 10.3389/fpls.2025.1675432

**Published:** 2025-10-06

**Authors:** Zhaochu Wang, Xinyu Xu, Peilan Zhang, Ye Huang, Chenzi Zhao, Yousry A. El-Kassaby, Yangtao Chen, Jing Wang, Rong Shi

**Affiliations:** ^1^ The Affiliated People’s Hospital of Fujian University of Traditional Chinese Medicine, Fuzhou, China; ^2^ College of Landscape Architecture and Art, Fujian Agriculture and Forestry University, Fuzhou, China; ^3^ Department of Forest and Conservation Sciences, Faculty of Forestry, The University of British Columbia, Vancouver, BC, Canada; ^4^ School of Tropical Agriculture and Forestry, Hainan University, Haikou, China

**Keywords:** *Bidens alba*, flavonoids, terpenoids, secondary metabolism, tissue specificity

## Abstract

**Introduction:**

*Bidens alba* is a traditional medicinal plant valued for its antioxidant, anti-inflammatory, and antimicrobial properties, largely attributed to flavonoids and terpenoids. However, the tissue-specific distribution and regulatory mechanisms of these metabolites remain poorly understood.

**Methods:**

We employed a combined approach of reference-guided transcriptomics and widely targeted metabolomics to investigate the biosynthesis and accumulation of flavonoids and terpenoids in four tissues (flowers, leaves, stems, and roots) of *B. alba*.

**Results:**

A total of 774 flavonoids and 311 terpenoids were identified. Flavonoids were enriched in aerial tissues, while certain sesquiterpenes and triterpenes accumulated in roots. Transcriptome profiling revealed tissue-specific expression of key biosynthetic genes, including *CHS, F3H, FLS, HMGR, FPPS*, and *GGPPS*, which corresponded with metabolite patterns. Several transcription factors, such as *BpMYB1, BpMYB2*, and *BpbHLH1*, were identified as candidates regulating flavonoid biosynthesis, with *BpMYB2* and *BpbHLH1* showing contrasting expression between flowers and leaves. For terpenoid biosynthesis, *BpTPS1, BpTPS2*, and *BpTPS3* were identified as putative regulators of enzymes including *GPPS* and *DXR*, with *BpTPS2/3* highly expressed in flowers.

**Discussion:**

These findings provide new insights into the transcriptional and metabolic basis of tissue-specific secondary metabolism in *B. alba*. The identified candidate genes and regulatory factors represent valuable targets for future functional validation and hold potential for medicinal development and molecular breeding of this species.

## 1 Introduction

*Bidens alba*, a member of the Asteraceae family, is a dicotyledonous herbaceous plant widely distributed in tropical and subtropical regions, with high prevalence in southern China. It commonly grows along roadsides, wastelands, and field margins. This species demonstrates strong environmental adaptability and competitive ecological traits, and has attracted considerable attention due to its diverse medicinal properties (Zhang et al., 2023). Traditionally, *B. alba* has been employed to treat a variety of ailments, including wounds, fever, jaundice, gastrointestinal discomfort, common cold, eye infections, urinary tract infections, and dermatological conditions (Ong et al., 2008; Widodo et al., 2024). In folk medicine, its leaves, stems, roots, and flowers are frequently processed into decoctions, poultices, or oral preparations, serving as complementary or alternative remedies for common and chronic diseases. In China, *B. alba* is used to manage inflammation, dysentery, hyperglycemia, pharyngitis, and intestinal infections (Bo et al., 2012). Its young shoots and leaves—either fresh or dried—are also used in herbal teas or as flavoring agents (Priestap et al., 2008), making it a valuable resource for both dietary and medicinal applications in humans and livestock.

Phytochemical investigations have shown that various tissues (root, stem, leaf, flower) of *B. alba* contain a broad range of secondary metabolites, including polyacetylenes, polyphenols, flavonoids, alkynes, alkaloids, phenolic acids, rudbeckins, phytosterols, chlorophylls and their derivatives (e.g., chlorophyll a and degradation products), terpenoids, and fatty acids (Edo et al., 2025b). Among these, flavonoids and terpenoids have been recognized as the principal bioactive constituents. Flavonoids, synthesized mainly through the phenylpropanoid and flavonoid biosynthetic pathways, are widely distributed secondary metabolites in higher plants. They contribute to oxidative stress mitigation, metal ion chelation, enzyme regulation, and membrane stabilization, playing crucial roles in plant responses to abiotic and biotic stresses (Agati et al., 2012; Panche et al., 2016). Pharmacologically, flavonoids exhibit diverse activities including antioxidant, anti-inflammatory, antitumor, anti-allergic, neuroprotective, and cardiovascular protective effects (Havsteen, 2002). Recent studies show that flavonoids are abundant across plant species. For instance, *Arabidopsis thaliana* flavonoids have been implicated in soil remediation (Hernández-Vega et al., 2024), while nitrogen availability significantly affects flavonoid accumulation and medicinal quality in *Epimedium pubescens* (Liu et al., 2025). Identified flavonoids in *B. alba* include quercetin, kaempferol, apigenin, and isorhamnetin, with some, such as okanin glycosides, considered chemotaxonomically and pharmacologically important (Javed et al., 2025). Moreover, ethanol extracts from aerial parts have been reported to suppress gastric secretion and pepsin activity, indicating anti-ulcer potential, likely linked to quercetin content (Shams and Eissa, 2022).

Terpenoids are synthesized via the mevalonate (MVA) and methylerythritol phosphate (MEP) pathways, and their structural diversity and tissue-specific distribution are often associated with developmental processes and environmental responses (Henry et al., 2018). For example, terpenoid biosynthesis in tea plants is regulated by alternative splicing (Jiang et al., 2023), contributes to flavor traits in *Brassica rapa* (Liu et al., 2023), and plays anti-inflammatory roles in triterpenoid-rich *Polygala tenuifolia* (Meng et al., 2023). Despite evidence of abundant flavonoids and terpenoids in *B. alba*, the molecular regulatory mechanisms governing their biosynthesis remain unclear, posing a major challenge for comprehensive resource utilization. In recent years, the integration of widely targeted metabolomics with high-throughput RNA sequencing (RNA-seq) has become a powerful approach to unravel the complex regulation of plant specialized metabolism. This combinatorial strategy enables simultaneous mapping of metabolite profiles and gene expression networks, providing insights into key biosynthetic pathways and transcriptional regulators (Nguyen et al., 2024). In medicinal plants, multi-omics analyses at the tissue level have been successfully used to decipher organ-specific accumulation and regulation of bioactive metabolites, and to support metabolic engineering efforts. For example, research on *Amomum tsao-ko* has revealed tissue-specific features of terpenoid biosynthesis (Chen et al., 2025).

In this study, we selected *B. alba* as a model to investigate the tissue-specific distribution and molecular basis of flavonoid and terpenoid biosynthesis. Samples from leaves, stems, flowers, and roots were collected and subjected to integrated metabolomic and transcriptomic analyses. The goal was to systematically profile metabolite accumulation patterns and identify key biosynthetic genes across different organs. Correlation analyses between metabolite levels and gene expression, along with qRT-PCR validation, were conducted to confirm the tissue-specific expression of major biosynthetic genes. Ultimately, this work aims to elucidate the regulatory framework underlying flavonoid and terpenoid biosynthesis in *B. alba*, thereby advancing our understanding of its secondary metabolism and offering a theoretical foundation for its medicinal development and resource utilization.

## 2 Materials and methods

### 2.1 Plant materials, tissue collection and RNA extraction and sequencing

Different tissues (leaf, stem, root, flower) of *B. alba* were collected from healthy, uniformly growing plants (n = 5) on the campus of Fujian Agriculture and Forestry University (Fuzhou, China). All tissues were immediately frozen in liquid nitrogen after harvest and stored at –80°C until further use. Total RNA was extracted using the FastPure Universal Plant Total RNA Isolation Kit (Wang et al., 2024), and quality was assessed prior to sequencing. RNA-seq libraries were constructed using the VAHTS Universal V6 RNA-seq Library Prep Kit for MGI^®^ (#NRM605), and sequencing was performed on the DNBSEQ-T7 platform by Benagen (Wuhan, China).

### 2.2 Widely targeted metabolomics analysis

Tissue samples were ground to a fine powder and extracted using 70% methanol containing internal standards. Metabolite profiling was carried out using ultra-performance liquid chromatography (UPLC, ExionLC™ AD) coupled with tandem mass spectrometry (MS/MS). Metabolite identification and quantification were performed using a self-built local database, and raw MS data were processed using Analyst 1.6.3 software (Sima et al., 2025). Principal component analysis (PCA) was conducted using the prcomp function in R after unit variance scaling (Patterson et al., 2006). Hierarchical cluster analysis (HCA) and Pearson correlation coefficient (PCC) analyses were performed using the ComplexHeatmap package, and results were visualized as heatmaps. Differential metabolites were screened based on variable importance in projection (VIP > 1) from OPLS-DA models and |Log_2_ fold change| ≥ 1. Score plots and permutation tests were visualized using the MetaboAnalystR package. Annotated metabolites were mapped to KEGG pathways (http://www.kegg.jp) for enrichment analysis.

### 2.3 Transcriptome analysis

Raw sequencing reads were quality filtered and mapped to the reference genome of *B. alba* (https://zenodo.org/records/10160015). Functional annotations were integrated from seven public databases: Nr, Pfam, Uniprot, KEGG, GO, KOG/COG, and PATHWAY. Functional information was obtained by combining homology-based annotation and protein domain prediction. Protein sequences translated from transcripts were aligned to the Uniprot and Nr databases using Diamond (v2.0.6) (Buchfink et al., 2015) with an E-value cutoff of 1e^-5^. GO terms were assigned using Uniprot-GOA mapping, and KOG/COG categories were also annotated. Conserved protein domains and motifs were identified by hmmscan (v3.3.2) (Wilde et al., 2023) against Pfam and KOfam databases. Gene expression levels were calculated as FPKM using RSEM, and differential expression analysis was performed using DESeq2 (Love et al., 2014). Heatmaps of genes with FPKM > 0.5 were generated using TBtools (v2.142) (Chen et al., 2023). Transcription factors were identified using the PlantTFDB (https://www.hsls.pitt.edu/obrc/index.php?page=URL1208279124). Protein–protein interaction (PPI) networks for differentially expressed genes were constructed using the STRING database (https://cn.string-db.org/). For species not available in STRING, BLASTP alignment was used to map proteins to the closest available species in the database for network reconstruction.

### 2.4 qRT-PCR validation

Twelve key genes involved in flavonoid and terpenoid biosynthetic pathways showing high expression in specific tissues (root, stem, leaf, or flower) were selected for quantitative real-time PCR (qRT-PCR) validation. cDNA was synthesized using the ABScript Neo RT Master Mix for qPCR with gDNA Remover. Gene-specific primers were designed using Primer Premier 6 (**Supplementary Table S1**) (Wang et al., 2017). GAPDH was used as the internal reference. Reactions were performed with 2X Universal SYBR Green Fast qPCR Mix using three biological replicates per sample. The thermal cycling conditions were: 95 °C for 3min, followed by 45 cycles of 95 °C for 10 s and 60 °C for 35 s. Relative expression levels were calculated using the 2^–ΔΔCt method. Statistical analyses were conducted using SPSS v25.0 (Yang et al., 2022), and graphs were generated with Origin 2019 (v9.6.0.172).

## 3 Results

### 3.1 Metabolomic profiling of different tissues in *Bidens alba*

**Figure 1:**
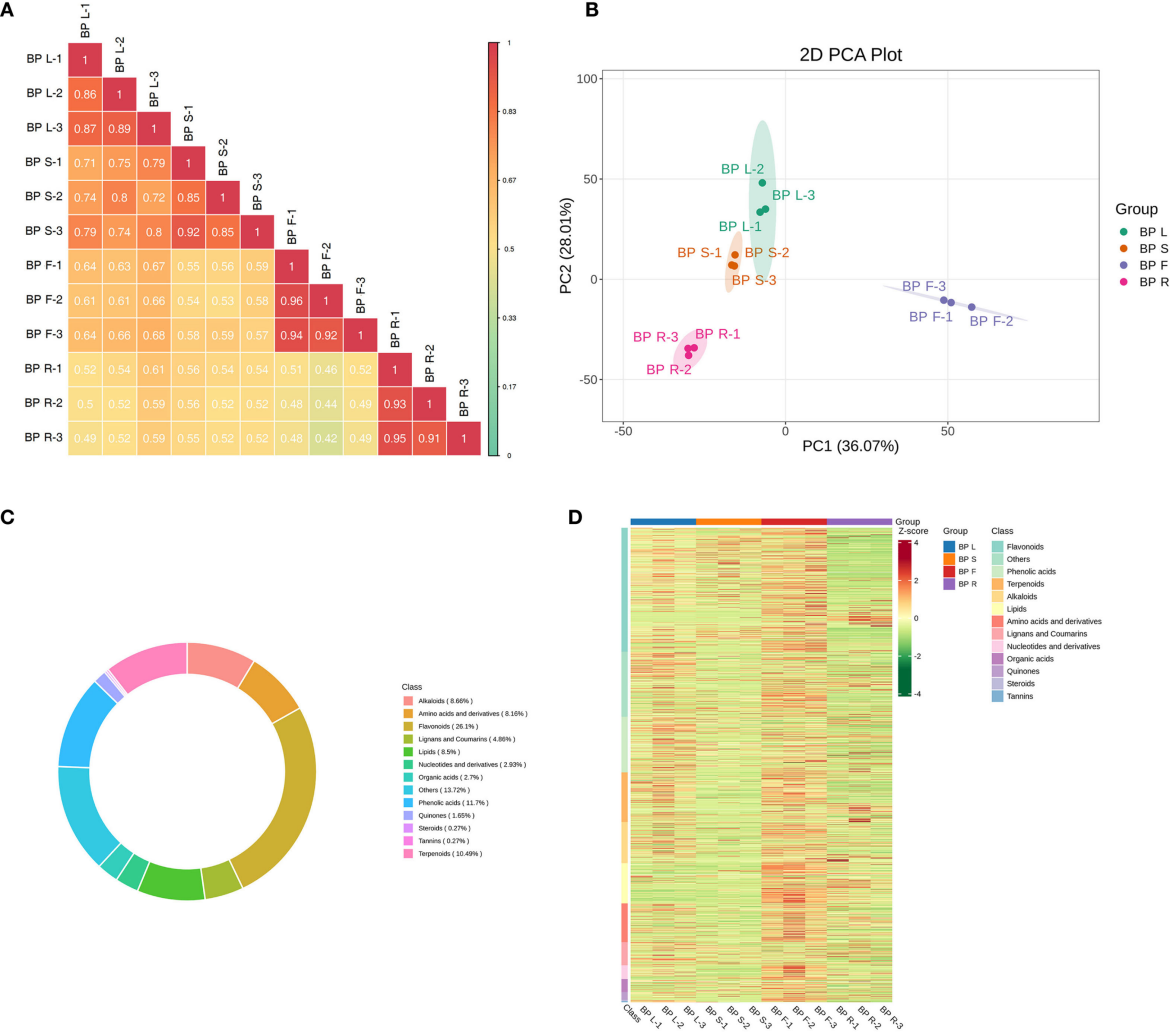
Widely targeted metabolomic profiling of different tissues of *B*. *alba*. **(A)** Correlation heatmap of biological replicates from each tissue. **(B)** Principal component analysis (PCA) of metabolite profiles. **(C)** Classification and proportion of identified metabolites. **(D)** Heatmap showing hierarchical clustering of metabolite accumulation across tissues.

To investigate the metabolic differences and potential medicinal value among different tissues of the medicinal plant *B. alba*, a widely targeted metabolomics approach was employed to systematically analyze four tissues: leaves (BPL), stems (BPS), flowers (BPF), and roots (BPR). Correlation analysis revealed high consistency among biological replicates within the same tissue (correlation coefficient > 0.9), indicating the reliability of the data (**Figure 1A**). Principal component analysis (PCA) further demonstrated significant separation among different tissues at the metabolomic level, with PC1 and PC2 explaining a total of 64.08% of the variance (**Figure 1B**). The four tissue types—BPL, BPS, BPF, and BPR—clustered separately in the two-dimensional space without overlap. Notably, flower and root samples were the most distant along the PC1 axis, suggesting the greatest metabolic differences between these tissues. These results highlight the presence of distinct tissue-specific metabolic profiles in *B. alba*.

Metabolite classification statistics showed that flavonoids (26.1%), phenolic acids (11.7%), and terpenoids (10.49%) constituted the most abundant classes, suggesting potential antioxidant and anti-inflammatory properties (**Figure 1C**). Furthermore, heatmap analysis revealed distinct patterns of metabolite accumulation among tissues, especially for representative groups of secondary metabolites such as flavonoids, phenolic acids, terpenoids, and alkaloids, which exhibited tissue-specific expression profiles (**Figure 1D**). These findings comprehensively reveal the metabolic characteristics of different tissues in *B. alba* and provide a theoretical basis for further investigation into the accumulation patterns of its medicinally active compounds.

### 3.2 Tissue-specific metabolic differences of flavonoids and terpenoids in *B. alba*

**Figure 2:**
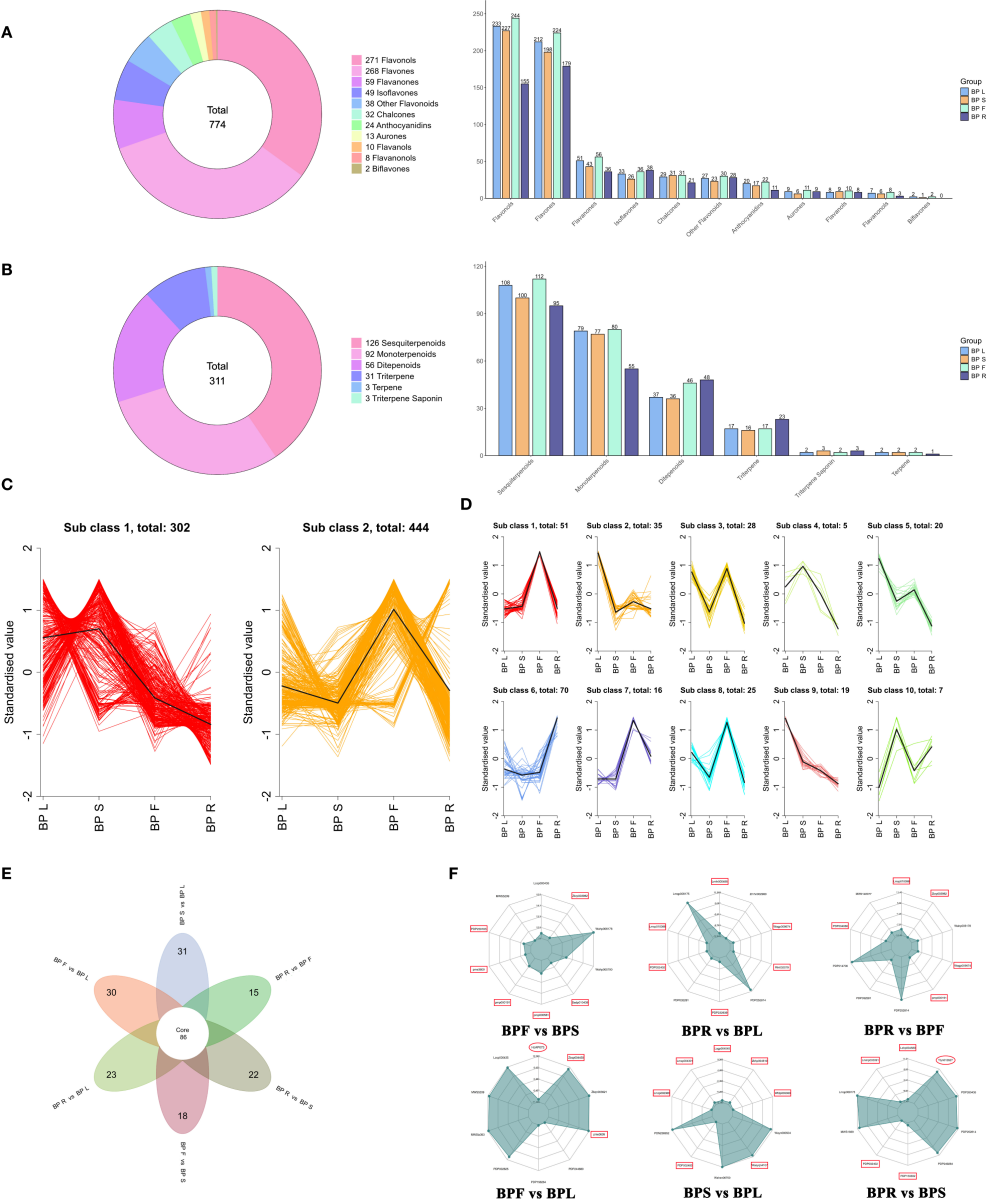
Differential flavonoid and terpenoid metabolites in different tissues of *B*. *alba*. **(A)** Number of flavonoid metabolites. **(B)** Number of terpenoid metabolites. **(C)** K-means clustering of flavonoid metabolites. **(D)** K-means clustering of terpenoid metabolites. **(E)** Venn diagram. **(F)** Top 10 differential metabolites in different comparison groups (flavonoids are marked with rectangles, terpenoids with ellipses).

Flavonoids and terpenoids are key classes of secondary metabolites with important pharmacological functions in medicinal plants. To further elucidate the tissue-specific accumulation of these compounds in *B. alba*, we systematically analyzed the composition and distribution of flavonoid and terpenoid subclasses across different plant organs (**Figures 2A, B**). A total of 774 flavonoids were identified, with flavonols and flavones being the predominant subclasses. Among these, the floral tissues exhibited the highest diversity, particularly in flavonols (244 compounds) and flavones (224 compounds), followed by leaves (BPL), while roots (BPR) had comparatively fewer flavonoid types. Terpenoid profiling revealed 311 terpenoid compounds, with sesquiterpenoids being the major class. Notably, sesquiterpenoids accumulated abundantly in both flowers (BPF) and roots (BPR), whereas triterpenoids (23 compounds) were predominantly enriched in roots, indicating that the root is likely a key site for the biosynthesis and accumulation of triterpenoids.

These findings highlight a clear tissue-specific metabolic pattern in *B. alba*. Further pairwise comparisons among six tissue groups (BPF vs. BPS, BPR vs. BPL, BPR vs. BPF, BPL vs. BPF, BPS vs. BPL, and BPR vs. BPS) revealed numerous differentially accumulated metabolites (DAMs). To better understand the trends in metabolite abundance, K-means clustering was performed on UV-scaled relative abundances of identified flavonoids and terpenoids (**Figures 2C, D**; **Supplementary Table S2**). Two major accumulation trends were observed for flavonoids: one cluster exhibited high levels in stems (BPS), while another showed enrichment in flowers (BPF). Terpenoids were grouped into eight distinct accumulation patterns across tissues. Of particular note, several sesquiterpenoids showed preferential accumulation in roots (BPR). Venn diagram analysis (**Figure 2E**) identified 86 core metabolites consistently different across all comparison groups, indicating their potential involvement in common regulatory or metabolic differentiation processes. Additionally, several tissue-specific DAMs were found, such as 31 unique to the BPS vs. BPL group and 30 unique to the BPF vs. BPL group, underscoring the strong organ-specific specialization in secondary metabolism. These core and tissue-specific metabolites offer valuable targets for future pathway reconstruction and functional investigation.

Further analysis of the top 10 significantly altered metabolites in the BPF vs. BPS comparison (**Figure 2F**; **Supplementary Table S3**) revealed a predominance of flavonoids, including anthocyanins, isoflavones, aurones, and flavones such as cyanidin and its derivative (pme3609), and apigenin-7-O-glucoside (pmp000581). Some alkaloids and amino acid derivatives were also significantly upregulated, indicating tissue-specific differences in flavonoid biosynthesis and transport between flowers and stems. In the BPR vs. BPL comparison, most DAMs were flavonoids (e.g., flavonols, chalcones, and dihydroflavonoids), all of which were downregulated in roots, along with significant differences in phenolic acids, alkaloids, and quinones. The BPR vs. BPF comparison showed consistent downregulation of several flavonoids, including dihydroflavonoids, chalcone isoflavones, and aurones, while amino acid derivatives and alkaloids were differentially expressed, reflecting distinct metabolic activities in roots versus flowers. In the BPF vs. BPL comparison, flavonoids (especially flavones and anthocyanins) were significantly upregulated in flowers, while triterpenoids such as ganoderic aldehyde C (HJAP073) were more abundant in roots. Other notable DAMs included amino acid derivatives, lignans, and coumarins. In the BPS vs. BPL comparison, differential flavonoids included dihydroflavonoids, flavonols, and flavones (e.g., Isoetin 7-glucoside), along with a small number of organic acids and lipids. Quercetin-3-O-arabinoside-glucoside (Wasyqn4107) showed the most significant difference, with most flavonoids downregulated in stems compared to leaves. In the BPR vs. BPS comparison, significant DAMs included flavonols and flavones (downregulated), while triterpenoids such as sarasinoside (Ylyn012627), along with phenolic acids, lipids, and alkaloids, were upregulated. Notably, sarasinoside was one of the most significantly altered triterpenoids in this comparison.

### 3.3 Transcriptome-based analysis of differential gene expression in different tissues of *B. alba*

To investigate transcriptional differences among tissues of *B. alba*, we first evaluated the quality of raw transcriptome sequencing data. The results showed that the Q20 base proportion for all samples exceeded 98.25%, Q30 exceeded 94.37%, and the GC content ranged from 43.08% to 43.96%, indicating high-quality sequencing data. After filtering, each sample yielded 6.41–6.45 Gb of clean data (**Supplementary Table S4**). The clean reads were then mapped to the *Bidens alba* reference genome, with mapping rates above 90.36% for all samples (**Supplementary Table S4**), further confirming the reliability and absence of contamination in the dataset, suitable for downstream differential expression analysis.

**Figure 3:**
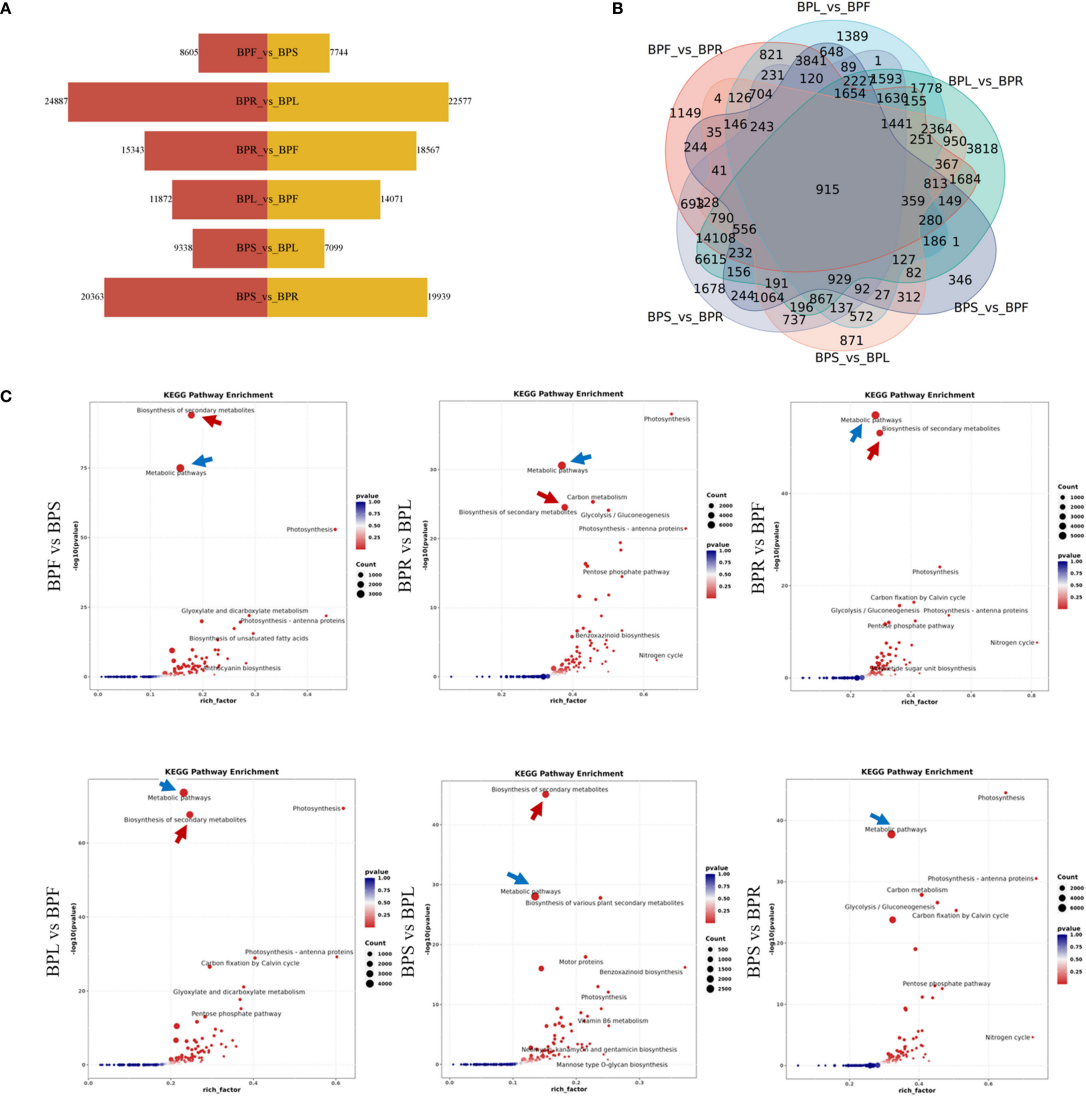
Expression patterns of differentially expressed genes **(A)** Number of differentially expressed genes. **(B)** Venn diagram. **(C)** KEGG pathway enrichment.

Based on this, differential gene expression (DEG) analysis was conducted using DESeq2, with screening criteria set as |log2FoldChange| ≥ 1 and padj ≤ 0.05. Among the six pairwise tissue comparisons (**Figure 3A**), the largest number of DEGs was detected between root and leaf (BPR vs BPL), totaling 47,464 DEGs, including 22,577 upregulated and 24,887 downregulated genes. This was followed by stem vs root (BPS vs BPR), with 40,302 DEGs (19,939 upregulated, 20,363 downregulated). In contrast, fewer DEGs were observed between flower vs stem (BPF vs BPS) and stem vs leaf (BPS vs BPL), with 16,349 and 16,437 DEGs, respectively. Additionally, Venn diagram analysis (**Figure 3B**) revealed 915 commonly differentially expressed genes across all six comparisons, suggesting that these genes may be involved in coordinated regulation of metabolic processes or maintenance of tissue-specific expression, potentially serving as core regulators. These results indicate that gene expression differences are most pronounced between root tissue and leaf/stem, while stem and leaf/flower exhibit more similar expression profiles. To further explore the biological functions of these DEGs, KEGG pathway enrichment analysis was performed for all comparison groups (**Figure 3C**). The results showed that DEGs were mainly enriched in secondary metabolite biosynthesis (indicated by red arrows) and signal transduction pathways (indicated by blue arrows) across all comparisons. Notably, signal transduction pathways were most significantly enriched in the BPS vs BPR group. This suggests that tissue-specific functional differentiation primarily reflects differences in metabolic activity and signal response, with root tissue likely playing a central role in metabolic regulation.

In summary, *B. alba* exhibits significant transcriptional divergence among tissues, with the most distinct differences observed between root and other organs. The DEGs are mainly enriched in metabolic pathways, particularly those related to secondary metabolism and signal transduction, highlighting the functional and physiological differentiation among tissues.

### 3.4 Correlation analysis between DAMs and DEGs

**Figure 4:**
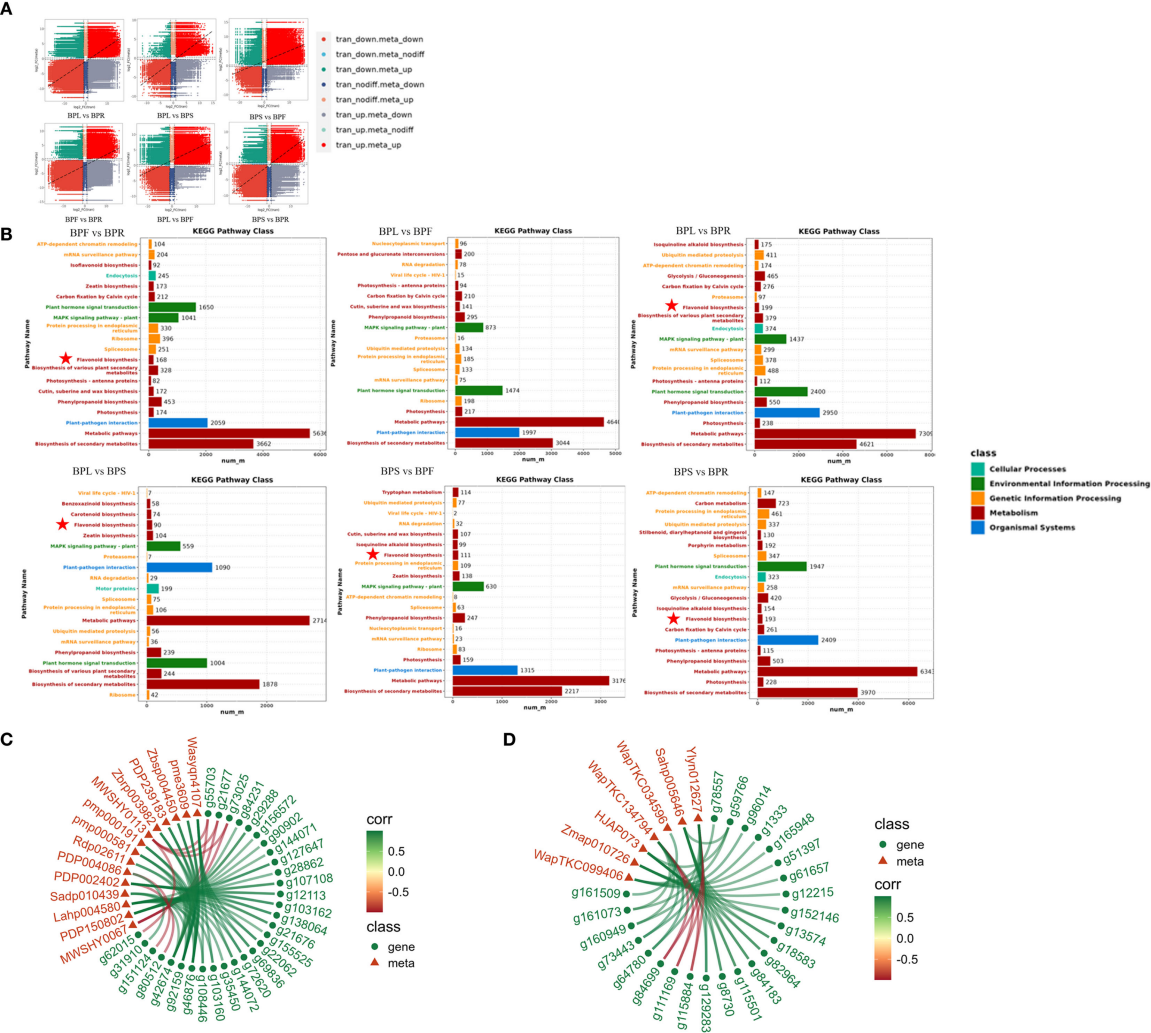
Correlation analysis between DAMs and DEGs **(A)** Nine-quadrant plot. **(B)** Combined KEGG enrichment analysis. **(C)** Flavonoid-related correlation network diagram. **(D)** Terpenoid-related correlation network diagram.

Differentially expressed genes (DEGs), by encoding key enzymes involved in metabolic processes, directly or indirectly affect the synthesis, accumulation, and transport of metabolites, thereby contributing to the metabolic differences among plant tissues. The transcriptome–metabolome nine-quadrant plots illustrated the trends of gene expression and metabolite accumulation across six tissue comparisons, showing a large number of genes and metabolites displaying coordinated upregulation (red dots) or downregulation (green dots). This indicates that transcriptional changes significantly impact metabolite accumulation, with a strong consistency in regulatory patterns between different tissues. For instance, in comparisons between leaves (BPL) and roots (BPR) or stems (BPS), upregulated and downregulated genes and metabolites were concentrated in their corresponding quadrants, reflecting tissue-specific regulatory modes. Meanwhile, some genes and metabolites exhibited discordant expression patterns (blue and gray dots), suggesting the presence of more complex regulatory mechanisms or spatiotemporal differences between transcriptional regulation and metabolite accumulation (**Figure 4A**).

KEGG pathway enrichment bar plots further confirmed this observation (**Figure 4B**). In all pairwise comparisons, the differentially expressed genes and metabolites were significantly enriched in metabolism-related pathways, especially secondary metabolite biosynthesis and primary metabolic pathways (e.g., carbon metabolism and amino acid metabolism), underscoring their central roles in tissue functional differentiation and metabolic specialization in *B. alba*. In contrast, pathways related to genetic information processing and environmental signal transduction were less enriched, highlighting the dominant contribution of metabolic regulation to tissue-specific differences. Notably, flavonoid biosynthesis pathways were significantly enriched in all comparisons except between leaves and flowers (BPL vs BPF), with the strongest enrichment observed between flowers and roots (BPF vs BPR). This suggests that the flavonoid pathway and its associated differential genes and metabolites are among the key contributors to tissue-specific metabolic variation, whereas terpene biosynthesis pathways appear to play a less dominant role across different tissues. To further validate this, we conducted correlation analyses of the key DEGs and differential metabolites in the flavonoid and terpene pathways (**Figures 4C, D**; **Supplementary Table S5**). For flavonoids, Hyperin (MWSHY0067) was strongly correlated with *CHI* (g55703), *4CL* (g21677, g73025), and *PAL* (g62015), while Rutin (MWSHY0067) was mainly associated with upregulation of *CHI* (g55703) and *4CL* (g21677, g73025). For terpenes, salaspermic acid (Ylyn012627) and Ishwarol (WapTKC134794) were highly correlated with *HDS* (g84699), *FPPS* (g111169), and *DXS* (g115884).

In summary, the metabolic differences among tissues of *B. alba* are predominantly driven by transcriptional regulation. Metabolic and secondary metabolite biosynthetic pathways serve as the core of functional divergence among plant organs, reflecting the organ-specific regulatory mechanisms underlying the synthesis and accumulation of bioactive compounds.

### 3.5 Identification of key genes involved in flavonoid and terpenoid biosynthesis across different tissues of *B. alba*

**Figure 5:**
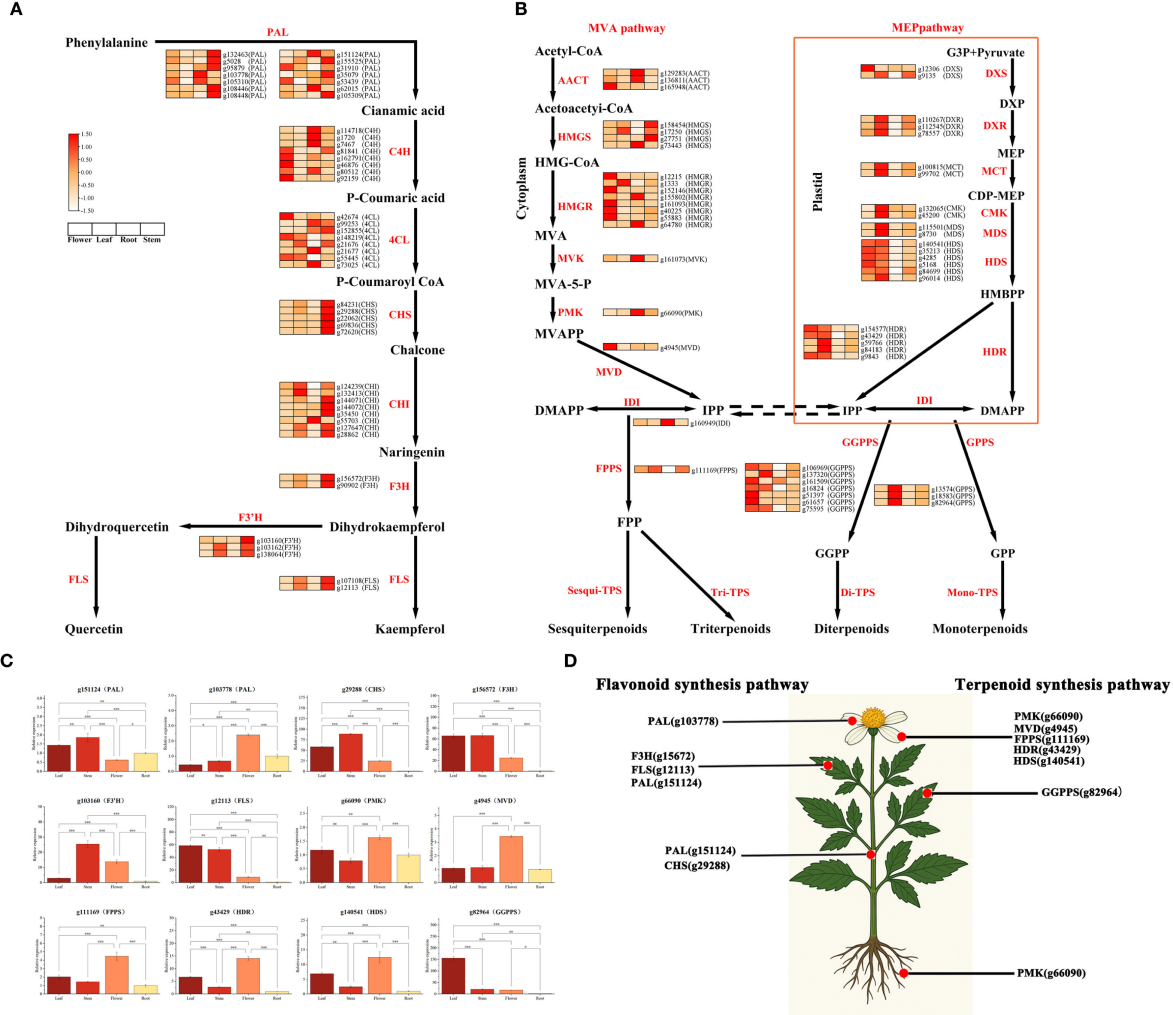
Flavonoid and terpenoid biosynthesis pathways **(A)** Flavonoid biosynthetic pathway. **(B)** Terpenoid biosynthetic pathway. **(C)** qRT-PCR validation of key biosynthetic genes. **(D)** Tissue-specific expression patterns of key genes across different tissues.

To gain insights into the tissue-specific biosynthetic mechanisms of flavonoids and terpenoids in *Bidens alba*, we performed an integrative analysis combining transcriptomic and widely targeted metabolomic data. This approach enabled the identification of key enzyme-encoding genes within relevant metabolic pathways and their expression profiles across different tissues. As illustrated in **Figure 5A**, **Supplementary Table S5**, heatmap analysis of flavonoid biosynthetic genes—including *PAL*, *C4H*, *4CL*, *CHS*, *CHI*, *F3H*, and *FLS*—revealed distinct expression patterns across flowers, leaves, stems, and roots. Notably, most of these genes exhibited elevated transcript levels in stems and leaves. Several gene copies of *PAL*, *CHS*, and *CHI*, which are involved in the early steps of flavonoid backbone formation, were significantly upregulated in stems, suggesting their central roles in initiating flavonoid biosynthesis. Moreover, downstream genes such as *F3H* and *FLS*, responsible for structural modification, were highly expressed in flowers, stems, and leaves, consistent with the observed accumulation of flavonol derivatives in these tissues based on metabolomic data. Regarding terpenoid biosynthesis (**Figure 5B**), genes from both the mevalonate (MVA) pathway in the cytosol and the 2-C-methyl-D-erythritol 4-phosphate (MEP) pathway in plastids showed distinct tissue-specific expression profiles. Key MVA pathway genes such as *HMGR*, *MVK*, *PMK*, and *FPPS* were predominantly expressed in flowers, indicating their involvement in the biosynthesis of sesquiterpenoids and triterpenoids in floral tissues. Conversely, MEP pathway genes—including *DXS*, *DXR*, *MCT*, *CMK*, *HDS*, *HDR*, and *IDI*—were more active in leaves, suggesting plastidial monoterpenoid biosynthesis is primarily leaf-associated. Importantly, genes encoding prenyltransferases like FPPS, GGPPS, and *GPPS*, which catalyze the formation of precursors *FPP*, *GGPP*, and *GPP*, respectively, were expressed in multiple tissues, supporting the plant’s capacity to produce diverse terpenoid subclasses. These expression patterns strongly align with metabolite distribution, further confirming the integrative results.

To validate transcriptomic findings, we selected 13 key structural genes involved in flavonoid and terpenoid biosynthesis for qRT-PCR analysis (**Figure 5C**). Most genes displayed significant tissue-specific expression differences (P < 0.05 or P < 0.001), closely mirroring transcriptome-derived trends. For flavonoid biosynthesis, genes such as *PAL* (g151124, g103778), *CHS* (g29288), *F3H* (g156572), *FLS* (g12113), and *F3’H* (g103160) were markedly upregulated in stems and leaves, with F3H and FLS reaching peak expression in leaves—implying their crucial roles in tissue-specific flavonoid accumulation. For terpenoid metabolism, genes including *PMK* (g66090), *MVD* (g4945), *HDR* (g43429), *HDS* (g140541), and *GGPPS* (g82964) exhibited significantly higher expression in flowers (P < 0.001), with *GGPPS* showing the strongest floral enrichment, suggesting flowers as major sites of terpenoid biosynthesis. Additionally, *FPPS* (g111169) was highly expressed in floral tissue, indicating its involvement in precursor synthesis for sesquiterpenoids and triterpenoids. Overall, these results reveal that key structural genes in flavonoid and terpenoid pathways are expressed in a highly tissue-specific manner in *Bidens alba*. The spatial expression of these genes corresponds closely with the distribution patterns of their respective metabolites, highlighting the transcriptional regulation underlying the tissue-specific biosynthesis of medicinally relevant secondary metabolites (**Figure 5D**).

### 3.6 Analysis of transcription factors related to flavonoid and terpenoid biosynthesis in *B. alba*

**Figure 6:**
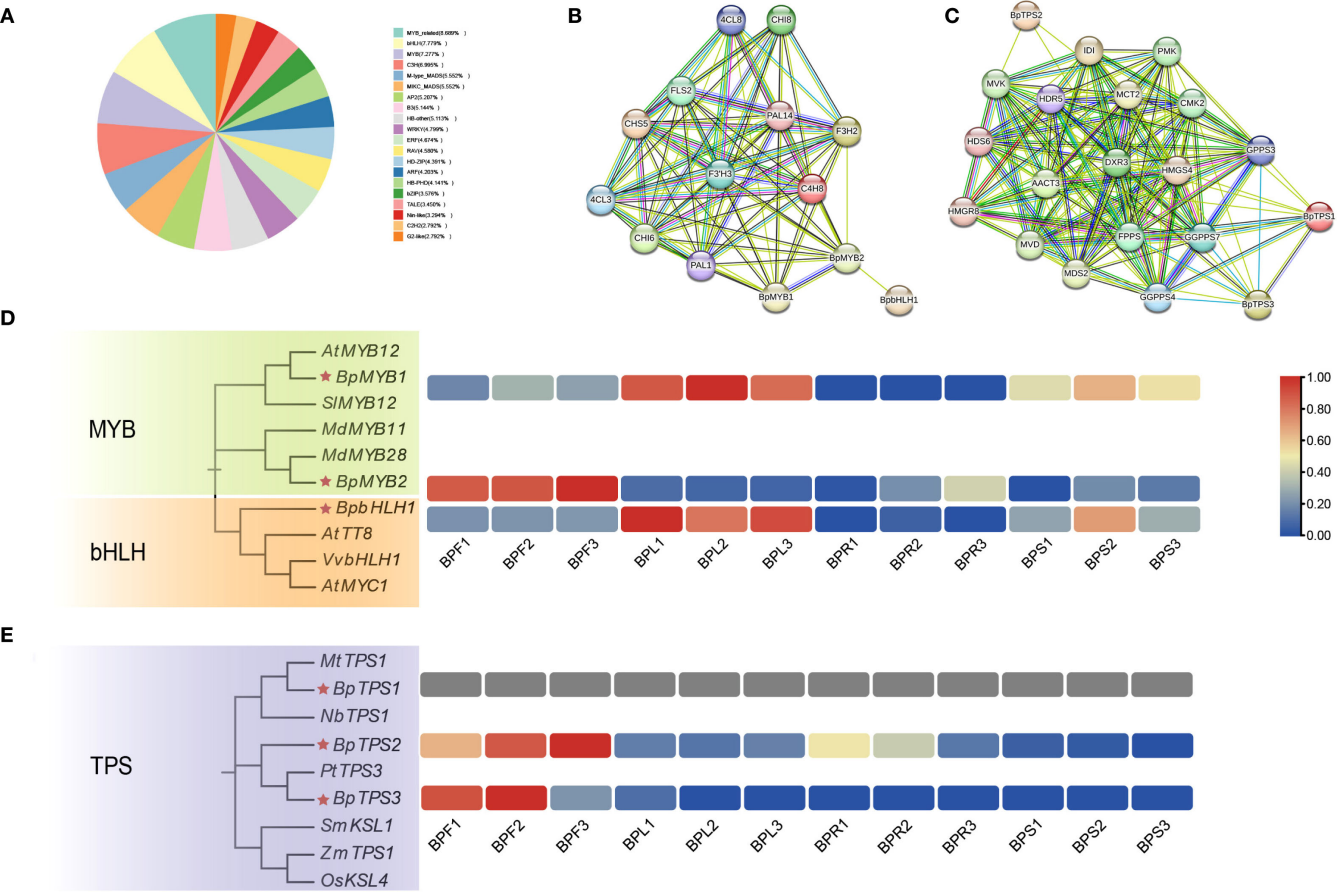
Transcription factors related to flavonoid and terpenoid biosynthesis **(A)** Proportion of transcription factor families identified in *B*. *alba*. **(B)** Protein–protein interaction network of transcription factors related to flavonoid biosynthesis. **(C)** Protein–protein interaction network of transcription factors related to terpenoid biosynthesis. **(D)** Expression patterns of MYB and bHLH family genes involved in flavonoid biosynthesis. **(E)** Expression patterns of TPS (terpene synthase) genes involved in terpenoid biosynthesis.

To investigate the transcriptional regulation of flavonoid and terpenoid biosynthesis in *B.alba*, the transcriptome sequences were annotated using the Plant Transcription Factor Database (PlantTFDB), resulting in the identification of 4,018 transcription factors (TFs) across 53 TF families. Among these, the MYB family accounted for 15.97%, MADS-box for 11.10%, and bHLH for 7.78% (**Figure 6A**), suggesting that these three families may play central regulatory roles in the biosynthesis of flavonoids and terpenoids in *B. alba*. Based on previous studies, we selected five experimentally validated transcription factors involved in secondary metabolite biosynthesis as query sequences: *MdMYB28* (accession: ATY37586.1), *AtMYB12* (AEC10843.1), *VvbHLH1* (XP_002270239.2), *MtTPS1* (AAV36464.1), and *PtTPS3* (AEI52903.1). Local BLAST analysis using these sequences identified six homologous transcription factors in *B. alba*: three associated with flavonoid biosynthesis—g35928 (*BpMYB1*), g74418 (*BpMYB2*), and g136574 (*BpbHLH1*)—and three associated with terpenoid biosynthesis—g154521 (*BpTPS1*), g144878 (*BpTPS2*), and g71866 (*BpTPS3*). Protein-protein interaction (PPI) networks were constructed to assess potential regulatory associations between these transcription factors and structural genes involved in flavonoid and terpenoid pathways (**Figure 6B**). *BpMYB1* and *BpMYB2* are putative regulators of key flavonoid pathway genes including *PAL*, *C4H*, *F3H*, *F3’H*, and *CHS*, suggesting these TFs play central roles in transcriptional activation. Although *BpbHLH1* did not directly interact with structural genes in the network, it was predicted to interact with *BpMYB2*, implying a possible cooperative or antagonistic role in regulation. For terpenoid biosynthesis (**Figure 6C**), *BpTPS1* and *BpTPS3* are putative regulators of several *GPPS* genes, suggesting their involvement in diterpenoid biosynthesis. Both genes also exhibited connections with other key enzymes such as *HMGS*, *DXR*, and *FPPS*. *BpTPS2* showed predicted interactions with *MVK*, *DXR*, and *IDI*, indicating its role in the MVA and MEP pathways.

To further investigate the evolutionary relationships of these candidate TFs, phylogenetic analysis was conducted using their amino acid sequences along with those of homologous genes from other species (accession numbers provided in **Supplementary Table 6**). Expression patterns of these TFs were then analyzed using FPKM-based heatmaps (**Figures 6D, E**). The heatmap revealed distinct tissue-specific expression of the six candidate genes. *BpMYB1* and *BpbHLH1* were highly expressed in leaves but showed negligible expression in flowers and roots, with only moderate expression in stems. Interestingly, *BpMYB2* displayed an opposite trend, being highly expressed in flowers while nearly absent in leaves. Combined with the PPI network, this suggests a potential antagonistic regulatory interaction between *BpMYB2* and *BpbHLH1* in different tissues. *BpTPS2* and *BpTPS3* were specifically and highly expressed in flowers, indicating strong tissue-specific expression patterns. These results collectively suggest that transcription factors from the MYB, bHLH, and TPS families are involved in organ-specific regulation of flavonoid and terpenoid biosynthesis in *B. alba*.

## 4 Discussion

*Bidens alba* is a medicinally valuable species widely used in traditional Chinese medicine. Among its diverse phytochemicals, flavonoids and terpenoids are considered key bioactive components. However, the full spectrum of these compounds, their biosynthetic pathways, and regulatory mechanisms remain largely unexplored. In this study, we systematically analyzed tissue-specific accumulation patterns of flavonoids and terpenoids in different organs (flowers, leaves, stems, and roots) of *B. alba*, and integrated transcriptomic data to uncover the expression patterns of key biosynthetic genes. These findings provide new insights into the plant’s specialized metabolism and offer molecular evidence to support its pharmacological potential.

### 4.1 Medicinal potential of flavonoids and terpenoids in *B.alba*

Flavonoids and terpenoids are widely distributed secondary metabolites in medicinal plants, known for their diverse pharmacological activities. Among plant-derived natural products, terpenoids represent one of the most structurally diverse classes, followed closely by flavonoids (Hao et al., 2024). In *B. alba*, a member of the Asteraceae family, more than 300 phytochemicals have been reported, including over 70 flavonoids and 30 terpenoids, which form the major basis for its bioactivity (Xuan and Khanh, 2016; Hodaei et al., 2018; Edo et al., 2025a). Our metabolomic analysis identified 774 flavonoid compounds and 311 terpenoids (**Figures 2A, B**; **Supplementary Table S1**). Importantly, these large numbers likely reflect the presence of multiple isomers and glycosylated derivatives derived from a smaller set of core aglycone backbones, a common feature observed in widely targeted metabolomics studies. Flavonols and flavones were the predominant flavonoid subclasses, while sesquiterpenes represented the most abundant terpenoid type. This chemical diversity highlights the plant’s high medicinal value and the potential for discovering novel bioactive compounds.

Tissue distribution analysis revealed that both metabolite classes predominantly accumulate in aboveground parts, especially flowers. Flavonoids, enriched in flowers and leaves, are known for antioxidant and antimicrobial properties, whereas triterpenoids tend to accumulate in roots and may contribute to anti-inflammatory and anticancer effects. Understanding the tissue-specific localization of these compounds can guide efficient harvesting strategies and targeted development of herbal products. Unlike *B. alba*, flavonoids in Scutellaria species (Lamiaceae) accumulate mainly in roots, and their terpenoids are often diterpenoid lactones (Yang et al., 2024). Similar co-occurrence of flavonoids and terpenoids has been documented in other medicinal species such as *Lonicera japonica*, *Ginkgo biloba*, and *Perilla frutescens* (Nishida and Satoh, 2004; Yuan et al., 2012; Hou et al., 2022). These observations underscore species-specific variations in metabolic distribution and suggest distinct regulatory mechanisms across taxa.

In traditional medicine, the whole plant is often used, potentially diluting the efficacy of active components. Clarifying the functional differentiation of chemical constituents among tissues facilitates precision harvesting and rational utilization of medicinal resources.

### 4.2 Tissue-specific differences in flavonoid and terpenoid profiles of *B.alba*

Beyond their pharmacological significance in human health, flavonoids and terpenoids play critical roles in plant development, stress responses, and ecological interactions such as pollinator attraction (Huang et al., 2022; He et al., 2023). In this study, substantial differences in metabolite profiles were observed across tissues. Key flavonoids included cyanidin, apigenin, kaempferol, luteolin, quercetin, naringenin, and genistein; terpenoids included lucialdehyde C and salaspermic acid (**Figure 2F**; **Supplementary Table S3**), many of which were previously reported in *B. alba* (Joshi et al., 2018; Malik et al., 2018; Adefegha et al., 2024). Several metabolites identified here have also been found in other plants, such as quercetin and kaempferol derivatives in raspberry (Yu et al., 2019), and rutin and apigenin in *Morus alba* (Hao et al., 2021). Lucialdehyde C from *Ganoderma lucidum* exhibits anti-cancer properties (Gao et al., 2002), while salaspermic acid from *Maytenus royleanus* also demonstrates notable bioactivities (Din et al., 2013).

Interestingly, this study identified multiple sesquiterpenoids in *B. alba* for the first time, including curcumenol (**Supplementary Table S2**), a compound originally isolated from *Curcuma wenyujin* with potent anticancer effects (Zhang et al., 2022). This may suggest convergent metabolic evolution with Zingiberaceae plants. Patchoulenol, typically derived from *Pogostemon cablin*, and orientalol F from *Alisma orientale*, both possessing broad pharmacological activities (Zahel et al., 2016; Wu et al., 2023), were also detected. These findings expand the known chemical repertoire of *B. alba*, indicating a broader biosynthetic capacity than previously recognized.

The detection of these novel compounds may result from advances in metabolomic sensitivity or inducible pathways activated by environmental stress. This reinforces the value of widely targeted metabolomics in non-model species for uncovering new secondary metabolites and enhancing our understanding of their therapeutic potential.

### 4.3 Tissue-specific expression of key genes in flavonoid and terpenoid biosynthesis

The biosynthetic pathways of flavonoids and terpenoids have been well-characterized in model plants, where key structural genes often exhibit strong spatiotemporal expression patterns in response to developmental or environmental cues. In *B. alba*, tissue-specific expression of differentially expressed genes was enriched in secondary metabolic pathways, particularly flavonoid biosynthesis, followed by terpenoid biosynthesis. Although numerous flavonoids and terpenoids have been reported in Bidens species, comprehensive analyses of their biosynthetic pathways and regulatory genes remain lacking. In contrast, extensive studies in other Asteraceae members such as *Chrysanthemum indicum*, *Carthamus tinctorius*, and *Xanthium sibiricum* have elucidated key genes involved in these pathways (Jiang et al., 2019; Wu et al., 2024; Li et al., 2025). This suggests the presence of conserved regulatory modules in Asteraceae that warrant further investigation in *B. alba*.

In this study, upstream flavonoid biosynthesis genes C4H and 4CL were broadly expressed across all tissues (**Figure 5A**; **Supplementary Table S5**), indicating ongoing flavonoid biosynthesis in multiple organs. These genes act as a metabolic bridge between primary metabolism (phenylalanine) and secondary metabolism, and are consistently upregulated in various medicinal plants such as *Echinacea angustifolia* (Chen et al., 2025) and *Areca catechu* (Lai et al., 2023). *CHS*, a rate-limiting enzyme in flavonoid biosynthesis, exhibited peak expression in stems, suggesting this tissue as a major site for flavonoid production. *CHS* expression has been positively correlated with total flavonoid levels in several species (Wang et al., 2018). Downstream enzymes such as *F3H*, *F3’5’H*, and *FLS* also showed high expression in stems, consistent with the accumulation patterns of quercetin, kaempferol, and their glycosides, confirming the coordination between gene expression and metabolite distribution. Although terpenoid biosynthesis showed less pronounced tissue specificity, clear patterns were still observed (**Figure 5B**; **Supplementary Table S5**). Genes involved in the MVA pathway, such as *HMGR*, *PMK*, and *FPPS*, were highly expressed in flowers, while genes from the MEP pathway, such as *DXS*, *HDR*, and *IDI*, were enriched in leaves. This suggests tissue-specific partitioning of sesquiterpene and monoterpene biosynthesis, corroborated by the respective accumulation of sesquiterpenes in flowers and triterpenes in roots. Similar trends have been reported in other medicinal plants such as *Panax ginseng* and *Salvia miltiorrhiza* (Yang et al., 2023). Precursor synthesis genes like *FPPS*, *GPPS*, and *GGPPS* showed consistently high expression across tissues, implying *B. alba* may possess the capacity for broad-spectrum terpenoid production, regulated by tissue-specific transcription factors or enzyme complexes.

Furthermore, qRT-PCR validation of genes such as *GGPPS* (g82964), *PAL* (g103778), and *FLS* (g12113) supported the transcriptomic data (**Figure 5C**). Notably, FPPS was highly expressed in flowers, possibly underlying the observed accumulation of sesquiterpenes, while *FLS* showed peak expression in leaves, aligning with the high levels of flavonols in this tissue. The stem, traditionally used in herbal medicine (Zahara et al., 2019), was found to be a metabolic hotspot for flavonoid biosynthesis, providing molecular evidence that bridges traditional use and modern pharmacological understanding. However, the accumulation of metabolites and the expression of biosynthetic genes may vary across developmental stages or under different environmental stresses. Future studies incorporating time-series sampling and stress-response experiments will therefore be crucial to expand and validate these findings. This work also provides a foundation for subsequent functional characterization of candidate genes and for their potential application in molecular breeding.

### 4.4 Transcriptional regulation of flavonoid and terpenoid biosynthesis in *B. alba*

In this study, several transcription factors potentially regulating flavonoid and terpenoid biosynthesis in *B. alba* were identified and functionally characterized using protein-protein interaction (PPI) networks and tissue-specific expression profiling. MYB and bHLH families are well-known regulators of secondary metabolism, particularly flavonoid biosynthesis, in many plant species (Xu et al., 2015). For instance, *AtMYB12* in Arabidopsis directly activates *CHS* and *FLS*, leading to increased flavonol accumulation (Wang et al., 2016), and *MdMYB28* in *Malus domestica* is reported to regulate upstream genes such as *PAL* and *CHS* (Ding et al., 2021). In *B. alba*, *BpMYB1* and *BpMYB2* showed strong interactions with *PAL*, *CHS*, and *F3H* (**Figures 6B, D**), suggesting they may coordinately regulate early flavonoid biosynthesis steps. Notably, *BpMYB1* and *BpbHLH1* were highly expressed in leaves, indicating a possible synergistic regulation, as previously proposed in other MYB-bHLH co-regulatory systems (Lloyd et al., 2017).

Regarding terpenoid biosynthesis, TPS (terpene synthase) genes play central roles in pathway regulation. Previous studies have shown that *PtTPS3* in *Populus trichocarpa* can activate genes involved in the MEP pathway, leading to diterpene production (Danner et al., 2011). In this study, *BpTPS1* and *BpTPS3* were found to interact with multiple precursor synthases including *GPPS*, *HMGR*, and *DXR*, suggesting their involvement in sesquiterpenoid or diterpenoid biosynthesis (**Figures 6C, E**). Tissue-specific expression revealed that *BpTPS2* and *BpTPS3* were highly expressed in floral tissues, a pattern also observed in *Salvia miltiorrhiza* TPS genes such as *SmTPS1* (Li et al., 2024), further supporting their potential regulatory function in flower-specific terpene accumulation. These findings provide novel insights into the transcriptional regulation of flavonoid and terpenoid metabolism in *B. alba* and offer candidate genes for future validation and potential metabolic engineering applications. Future studies using dual-luciferase assays, yeast one-hybrid, or transgenic approaches would help confirm these regulatory roles.

## 5 Conclusion

In this study, we systematically investigated the tissue-specific biosynthesis patterns of flavonoids and terpenoids in the medicinal plant *Bidens alba* by integrating transcriptomic and widely targeted metabolomic data. The results revealed that flavonoids were predominantly accumulated in aerial tissues, where key biosynthetic genes such as *PAL*, *CHS*, and *CHI* were significantly upregulated. In contrast, terpenoids—especially sesquiterpenes and triterpenes—were mainly enriched in flowers and roots, with corresponding genes in the MVA and MEP pathways (e.g., *HMGR*, *FPPS*, *GGPPS*) exhibiting strong tissue-specific expression. Correlation analysis between transcriptomic and metabolomic data further indicated that the differential accumulation of metabolites was largely driven by the transcriptional regulation of structural genes and was closely associated with specific pathways, including flavonoid and terpenoid biosynthesis. Notably, a set of transcription factors (TFs), MYB, bHLH, and TPS families, exhibited strong co-expression with key structural genes in a tissue-specific manner, suggesting that these TFs may act as upstream regulators modulating the spatial expression of secondary metabolic genes. Moreover, qRT-PCR validation confirmed the expression trends of several key genes, thereby enhancing the reliability of the transcriptomic results. Overall, this study provides new insights into the functional metabolic networks of *B. alba* and lays a theoretical foundation and offers candidate targets for future research on the biological functions of its active compounds and metabolic engineering. These findings are also valuable for the development and utilization of *B. alba* as a medicinal resource.

## Data Availability

The datasets presented in this study are publicly available. This data can be found here: https://www.ncbi.nlm.nih.gov/, accession number PRJNA1336433.
